# Recent advances in the use of imaging in psychiatry: functional magnetic resonance imaging of large-scale brain networks in late-life depression

**DOI:** 10.12688/f1000research.17399.1

**Published:** 2019-08-06

**Authors:** Kevin Manning, Lihong Wang, David Steffens

**Affiliations:** 1 Department of Psychiatry, University of Connecticut Health Center, Farmington, CT, USA

**Keywords:** neuroimaging, neuroscience, major depression, late-life depression, fMRI, executive control network, default mode network, salience network

## Abstract

Advances in neuroimaging have identified neural systems that contribute to clinical symptoms that occur across various psychiatric disorders. This transdiagnostic approach to understanding psychiatric illnesses may serve as a precise guide to identifying disease mechanisms and informing successful treatments. While this work is ongoing across multiple psychiatric disorders, in this article we emphasize recent findings pertaining to major depression in the elderly, or late-life depression (LLD), a common and debilitating neuropsychiatric illness. We discuss how neural functioning of three networks is linked to symptom presentation, illness course, and cognitive decline in LLD. These networks are (1) an executive control network responsible for complex cognitive processing, (2) a default mode network normally deactivated during cognitive demanding when individuals are at rest, and a (3) salience network relevant to attending to internal and external emotional and physiological sensations. We discuss how dysfunction in multiple networks contributes to common behavioral syndromes, and we present an overview of the cognitive control, default mode, and salience networks observed in LLD.

## Introduction

The practice of psychiatry has traditionally used a categorical approach to diagnosing psychiatric disorders. That is, in accord with the *Diagnostic and Statistical Manual of Mental Disorders* (DSM), a discrete psychiatric disorder is diagnosed when the patient’s behavioral signs and symptoms exceed a binary diagnostic rule or cut-point. For example, a patient meets criteria for a DSM-V diagnosis of major depression when at least five of nine symptoms are present for two weeks or more and at least one of the symptoms is depressed mood or loss of interest/pleasure. However, the categorical approach is limited when we consider the arbitrary nature of diagnostic cut-points and the multi-determined origins of behavior ^1, 2^. Thus, diagnoses based only on presenting symptoms may be quite varied in regard to the underlying pathophysiology ^2, 3^. Major depression in the elderly, for example, has a multitude of possible psychosocial or biological etiologies (or both), including decreased social support, endocrine abnormalities, inflammatory processes, vascular disease, and neurodegenerative illnesses ^4, 5^.

Novel dimensional conceptualizations of psychiatric illnesses have emerged to address the heterogeneity of traditional categorical diagnostic approaches. Thanks to advances in neuroimaging techniques, the fields of cognitive and affective neuroscience have helped identify neural systems that contribute to ubiquitous clinical symptoms across categorical psychiatric disorders ^6– 9^. This transdiagnostic approach to understanding psychiatric illnesses may serve as a precise guide to identifying disease mechanisms. However, continued research is needed to better understand transdiagnostic neurobehavioral systems. While this work is ongoing across multiple psychiatric disorders, we emphasize recent findings pertaining to major depression in older adults or late-life depression (LLD) in this article. We do so because LLD not only is common and is associated with detrimental clinical outcomes such as dementia ^10^, disability ^11^, and mortality ^12^ but also is challenging to treat. Thus, better understanding of the correlation between brain and behavior in LLD may inform understanding of the illness and identify new treatment targets. Below, we begin by providing an overview of the clinical significance of LLD. We then describe recent advances in the clinical correlates of brain-based networks in LLD.

## Clinical significance of late-life depression

LLD is a common and debilitating psychiatric disorder. Four percent of older women and three percent of older men have a current diagnosis of LLD ^5^, and LLD increases the risk of dementia ^10^, disability ^11^, and mortality ^12^. The risk of dementia is substantial in that LLD is associated with a four- to six-fold increase in the probability that Alzheimer’s disease or vascular dementia will be diagnosed ^10^. The detrimental consequences associated with LLD highlight the importance of facilitating timely diagnosis and adequate treatment. Yet, over 50% of patients do not respond to initial pharmacological treatment, and only one third of older patients who receive cognitive behavioral therapy or an antidepressant achieve remission from depression ^13^. Thus, novel depression treatments that can augment pharmacotherapy or be used independently in older adults with LLD are urgently needed. Neurobiologically informed treatment targets may hold promise in potentially altering the course of cognitive and affective symptoms in LLD as well as other psychiatric illnesses.

## Major neural networks relevant to neuropsychiatry

Neuroimaging has helped elucidate the brain regions and functions associated with psychiatric illnesses. Connectivity analyses from functional magnetic resonance imaging (fMRI) have been especially helpful. Functional connectivity (FC) refers to the phenomenon in which brain regions are activated and deactivated at the same time in the service of specific brain functions. A behavior or a clinical symptom typically involves synchronization of many brain regions in a network-based fashion. Evidence has identified three prominent functional networks in geriatric depression and other psychiatric illnesses ^14– 16^: (1) An executive control network (ECN), a functionally linked system made up of dorsolateral prefrontal, medial frontal, and lateral parietal cortices ^17^, is involved in complex cognition, particularly in executive control ^18^. (2) A default mode network (DMN), consisting of the medial prefrontal cortex, posterior cingulate cortex (PCC), inferior parietal cortex, and hippocampus, is normally deactivated during complex cognitive processing and active during internal mentation. (3) A salience/emotional processing network (SN), in which the right anterior insula and dorsal anterior cingulate cortex (dACC) are the primary hubs, assesses the significance of a stimulus and, together with amygdala activity, assigns emotional and motivational value to the stimulus.

Understanding FC abnormalities between brain regions or networks can help clarify clinical symptoms associated with neural substrates and identify potential treatment targets in LLD and prove useful in monitoring cognitive decline. In general, pronounced symptoms of major depression in the elderly, such as executive dysfunction, rumination, apathy, and negativity bias, are associated with decreased activity in the ECN and hyperactivity in the SN and DMN ^15, 16^. For example, (1) decreased FC within the ECN is related to worse executive function (EF) ^15, 19^ and worse cognitive reappraisal ability ^20^ or cognitive control over emotion ^21^; (2) increased FC within limbic regions, namely the DMN and SN, is associated with greater depression severity ^16, 22, 23^; (3) depression remitters exhibit increased FC within the ECN and decreased FC within DMN while non-remitters show no such changes ^15, 24^; (4) increased positive FC in the ECN-SN is related to worse EF and apathy in LLD ^16, 25^. Thus, interventions that enhance the efficiency and processing of the ECN and SN-DMN may be especially useful in the treatment of LLD.

## Network functioning in late-life depression

No single brain region is exclusively responsible for the heterogeneous symptoms of LLD. Instead, major depressive disorder likely arises when genetic and psychosocial predispositions drive structural and functional changes in multiple neural systems. Below, we have organized the subheadings of this review according to individual neural networks for ease of interpretation. Yet, as elaborated on below, it is clear that brain networks work in tandem to account for the diverse symptoms of LLD and other neuropsychiatric illnesses. Furthermore, although the primary emphasis of this review is on fMRI, multiple brain imaging techniques are increasingly used to understand the inner workings of disease states. The literature relevant to LLD and cognitive aging is noted below.

## The executive control network

Disruption of the ECN is prominent in LLD ^15, 26^. Clinically, disruption of the ECN results in executive dysfunction, including susceptibility to distraction, an inability to sustain attention, poor multi-tasking, organizational difficulties, and concrete or rigid thinking ^27^. Executive dysfunction is common in LLD. About 30 to 40% of non-demented older adults with LLD demonstrate impairments in EFs on neuropsychological examination ^28^. Depressed older adults often perform poorly on tests of word-list generation, cognitive flexibility and problem solving, planning, and susceptibility to interference or distraction ^29^ and performance on these tests correlates with brain abnormalities in prefrontal, medial frontal, and parietal cortices ^30^. Impaired executive control also predicts a poor illness course in LLD ^31^. Deficits in word-list generation and response inhibition predict poor and slow antidepressant response, relapse, and greater levels of functional disability ^32– 34^.

Depression and increasing age appear to exacerbate dysfunction of the ECN in LLD. Rao *et al*. ^35^ compared the effects of age and illness on cognitive processing by contrasting network activity in LLD (age of 65 or more) to age-matched non-depressed controls and young adults with major depression (age range of 18 to 33). During a cognitive control task within MRI, older adults with LLD exhibited increased activity in several ECN regions, as well as areas outside the ECN, compared with both age-matched controls and younger depression subjects. These findings suggested that areas of increased neural activation were due not to age or depression alone but to a combination of the two. Thus, Rao *et al*. ^35^ propose that ECN dysfunction in LLD might represent a type of accelerated aging process. This is consistent with the notion that depression precedes neurodegeneration and cognitive decline or places older adults at increased risk of those conditions. Also in keeping with this idea is the fact that although both younger and older adults with major depression exhibit greater cognitive impairments compared with their respective non-depressed age-matched peers, the magnitude of impairment is typically greater in the older adults ^36^.

Functional neuroimaging findings illustrate the role of the ECN in LLD. Using a seed-based correlation approach to explore resting-state FC (rsFC), Gandelman *et al*. ^37^ recently found that depression severity in 79 older adults with LLD was positively correlated with FC between the left dorsolateral prefrontal cortex (DLPFC) and other bilateral frontal regions (including the dACC). Furthermore, *greater* left DLPFC-dACC FC was associated with worse cognitive performance in LLD subjects, particularly in memory. Although these findings are consistent with a prior study by Alexopoulos *et al.* ^15^ documenting an association between ECN connectivity and executive dysfunction in LLD, findings from that study revealed that *decreased* FC of the ECN was associated with worse cognitive performance. Moreover, compared with remitters, non-remitters in the study by Alexopoulos *et al*. demonstrated decreased FC at baseline between key ECN regions, including the bilateral DLPFC, dACC, and inferior parietal cortices ^15^. Therefore, the association between worse cognitive performance and *greater* left DLPFC-dACC FC might initially appear counterintuitive. To help explain these findings, Gandelman *et al*. posit an interesting hypothesis whereby a bias toward negatively valenced stimuli may direct (increased) attention away from cognitive tasks at hand (thus also resulting in an increase in the FC of the ECN) ^37^. This novel hypothesis remains to be tested. Apparent FC irregularities in LLD may also simply reflect alternative ways in which patients process information. For example, Weisenbach *et al*. found that older adults with LLD exhibited *greater* activation in the left inferior frontal gyrus of the ECN during a word-list learning task when compared with non-depressed older adults, although there were no task behavioral differences between groups ^38^.

Non-pharmacological attempts to rehabilitate executive dysfunction and the ECN have become more common in older adults with or without depression. Computerized cognitive remediation is a treatment that has been used to improve cognition in the elderly. It is a behavioral intervention that uses controlled repetitive learning to improve cognitive functioning. A meta-analysis of 51 randomized controlled trials of computerized cognitive remediation in cognitively normal elderly patients indicates that cognitive training programs have a modest effect on global cognitive functioning ( *d* = 0.22) ^39^. However, cognitive training seems to work best in non-demented elderly patients with some degree of cognitive impairment, which is why it is especially promising to older adults with major depression. For example, a meta-analysis of 17 randomized controlled trials of computerized cognitive remediation in mild cognitive impairment found a moderate effect on multiple cognitive domains ( *g* = 0.35), including EF and memory ^40^, and the largest treatment-related cognitive gains in a small sample of LLD patients followed 4 to 6 weeks were found in those with the worst EF prior to treatment ^41^.

Evidence suggests that computerized cognitive remediation improves EF and depression symptoms in LLD as an augment to pharmacotherapy. Remediation practices may also modify FC. Morimoto *et al*. ^42^ tested computerized cognitive remediation in a small sample of LLD patients who were acutely depressed despite being on a long-standing variety of antidepressants and contrasted their depression and cognitive outcomes with those of 33 historical control patients who received escitalopram. Findings revealed that 30 training hours completed over 4 to 6 weeks improved response inhibition and cognitive flexibility and resulted in greater change in depression symptoms when compared with patients who received escitalopram. Questions remain regarding this intervention for older adults with major depression. The long-term effect of computerized cognitive training on depression and cognition has not been studied in LLD, and it is unclear whether the training can delay dementia risk. The impact of training on neural network functioning in LLD is also ambiguous, although studies have been conducted with non-depressed older adults. Specifically, cognitive training in a sample of 23 non-depressed older adults resulted in increased rsFC within the ECN and decreased rsFC within the SN; notably, changes correlated with improved cognition one year later ^43^.

## The default mode network

The DMN was originally described by Shulman *et al*. ^44^ and subsequently Raichle *et al*. ^45^, who observed correlated brain activity among certain brain regions during neuroimaging. These brain regions, now generally considered the ventral and dorsal medial prefrontal cortices, PCC/retrosplenial cortex, inferior parietal lobule, lateral temporal cortex, and hippocampal formation, increased metabolic activity during rest and decreased activity when engaged in cognitively demanding behavior (that is, that behavior associated with the ECN). Further investigation has found that DMN activity normally increases not during rest per se but during internal processes, such as perspective taking (for example, self-referential thinking and theory of mind) and in thinking about the past (for example, autobiographical memory) or the future ^46^.

Default mode activity is thought to be negatively correlated with activity within the ECN. The need to reduce the internal processes of the brain in order to focus on the (external) task at hand might be reflected by a reduction of DMN activity during effortful cognitive processing ^47^. Failure to reduce DMN activity might be a sign of an inability to quiet or inhibit internal mentation or emotional processing ^48^. This happens during both routine and complex activities. For example, Sheline *et al*. ^48^ found that depressed patients were less likely than non-depressed controls to decrease activity within the DMN when simply viewing negative pictures. Moreover, the DMN regions of the anterior cingulate and ventromedial prefrontal cortex failed to decrease in activity when depressed patients (but not non-depressed controls) attempted to reappraise (that is, think more positively about) negative pictures. Increased DMN activity is therefore associated with an inability to regulate emotions in major depression. Furthermore, the strength of the FC correlation between “top-down” brain control regions (for example, ECN) and “bottom-up” DMN/SN emotional centers is related to greater depression severity in LLD ^16, 25^.

Hyperactivity of the DMN may help drive the co-occurrence of anxiety in LLD. It was found that, when completing an MRI-based test of cognitive control, older adults with LLD and high anxiety, compared with older adults with LLD alone, experienced greater activation in the anterior and posterior regions of the DMN ^49^. One hypothesis is that DMN over-activity in anxious depression during cognitive demanding tasks could represent internal mentation interference (for example, rumination) ^49^, consistent with evidence that greater anxiety in older adults is associated with decreased connectivity between anterior and posterior hubs of the DMN ^50^. Alternatively, increased activation in posterior brain regions in anxious depression might represent heightened alertness to a threat in the environment ^22^.

Activity changes within the DMN occur with age and appear to precede cognitive decline. Lower baseline rsFC in the ECN, DMN, and SN is associated with cognitive decline in healthy elderly patients, even when structural volume loss is controlled for ^51^. Older adults with mild cognitive impairment, a clinical risk state for dementia, show lower FC in the PCC and medial temporal regions of the DMN, brain regions implicated in memory ^52^. In patients with actual dementia (Alzheimer’s disease), decreased activation within the DMN during rest and tasks is observed ^53, 54^, and some authors have found that reduced deactivation is related to increased amyloid burden, a presumptive biological marker of Alzheimer’s disease ^55, 56^. Notably, activation patterns within the DMN vary by Alzheimer’s disease stage. Findings from patients with early Alzheimer’s disease revealed hyperconnectivity in anterior medial frontal DMN regions and hypoconnectivity in posterior DMN (for example, PCC). Connectivity in both anterior and posterior DMN eventually decreases as dementia progresses ^57^. Some have speculated that anterior DMN over-activity in young adulthood occurs in the context of a physiological process that predisposes individuals to dementia in later life ^58^. This is clinically relevant to LLD, where increased DMN FC with the striatum (a reward center) is observed in patients who fail to respond to antidepressants when compared with patient responders ^59^. Furthermore, it has been found that DMN over-activity persists in some LLD patients compared with non-depressed controls even following patient remission from acute-state depression ^60^. Thus, DMN hyper-activity following depression treatment may be an indicator of later cognitive decline in LLD. This association between DMN over-activity and cognitive decline in LLD remains to be explored.

Besides FC, other fMRI analytic methods may help distinguish abnormal brain activity in LLD and mild cognitive impairment/pre-dementia states. Functional segregation (as opposed to FC-based functional integration) methods like regional homogeneity and amplitude of low-frequency fluctuation (ALFF) may be particularly helpful in deciphering the exact network node origin of abnormality. For example, compared with never-depressed older adults, older adults with LLD showed significantly increased ALFF in the middle temporal cortex, insula, fusiform gyrus, and cerebellum and significantly reduced ALFF in the inferior parietal cortex, mid-cingulate, and PCC/precuneus ^23^. Notably, in a coordinate-based meta-analysis of resting-state fMRI, the patients with amnestic mild cognitive impairment also show decreased ALFF in the PCC cortex ^61^. More research is needed to understand the biological overlap among patients with LLD and pre-dementia states.

## The salience network

The SN is important in the relationship of emotion to visceral function, allocation of attention, and orchestrating networks upon stimulus presentation ^62^. The right anterior insula is one of the primary SN nodes ^63^. Insula and amygdala activation can represent the value of emotional stimuli presented to the individual ^17^. The SN has prominent connections to other limbic areas and several subcortical structures (for example, amygdala and basal ganglia). The SN is a bottom-up processor that recruits other large-scale networks when responding to the salient stimulus ^17, 62^. For example, when attempting complex cognitive processing, the SN is thought to disengage the DMN and engage the ECN ^64^. Thus, at rest and in the absence of internal and external stimuli, FC between the SN and the DMN and ECN should typically be negatively correlated. Greater dissociations between the ECN and the SN correlate with better cognitive task performance ^65^, consistent with the ability to focus attention and ignore internal (for example, rumination) and external (for example, negative stimuli) distraction. This standard pattern of connectivity is disrupted in major depression ^66^. Older adults with LLD exhibit decreased negative FC between the ECN and the SN when compared with non-depressed controls ^67^. Furthermore, reduced negative FC between the ECN and the SN in LLD correlates with worse cognition ^67^, greater depression severity ^67^, and worse treatment response to antidepressants ^68^. Wang *et al*. ^25^ contrasted correlation patterns among the major neural networks in patients with LLD and non-depressed age-matched controls. Of note, the ECN and SN were correlated with an affective/default mode network, but this association was observed in non-depressed elderly patients only. No significant correlation was observed among elderly depressed subjects. These findings could represent a failure of inter-network cohesiveness in LLD.

Dysfunction to the SN is correlated with vegetative symptoms in LLD. Findings from one study found that, relative to LLD patients without apathy, LLD patients with high apathy had decreased rsFC of the right anterior insula with the right ACC, basal ganglia, and bilateral posterior parietal cortex ^16^. Findings from that study also revealed increased FC of the right insula to the right PCC in older adults with LLD and high apathy when compared with non-apathetic depressed elderly patients ^16^. Elsewhere, volumetric analyses reveal that older adults with major depression who exhibit prominent melancholia and vegetative symptoms have lower basal ganglia volumes compared with LLD patients without prominent melancholia and vegetative symptoms ^69^. Overall, one interpretation of these findings is that increased FC between SN and DMN may predispose individuals to depression, core features of which may include increased apathy and difficulty in engaging in cognitively demanding tasks while ignoring irrelevant, negative stimuli ^16^.

Increasing evidence also points to the importance of the SN in pre-dementia states. Increased within-SN FC has been found in cognitively normal individuals with elevated amyloid levels ^56, 70^, those carrying the apolipoprotein e4 risk allele for Alzheimer’s disease ^71^, patients with mild cognitive impairment ^72^, and individuals with clinically defined Alzheimer’s disease ^73^. Similar to the DMN FC, SN FC changes as a consequence of disease progression. Schultz *et al*. ^74^ recently conducted a study of 91 older adults who underwent resting-state fMRI and both amyloid (C Pittsburg compound B) and tau (AV1451) positron emission tomography (PET) imaging. Notably, increased connectivity in both the SN and DMN was associated with elevated amyloid imaging among participants with little evidence of tau. However, as both tau and amyloid levels increased, patients experienced decreased connectivity in the SN and DMN. These findings highlight the point that connectivity changes in preclinical dementia are not limited to the DMN. Given that depression severity is correlated with both DMN and SN functioning in LLD ^16, 22, 23^, severe depression may signal an intrinsic network dysfunction that heralds the clinical manifestation of cognitive impairment or the onset of eventual cognitive decline.

## Conclusions

Neural functions contribute to the complex symptom presentation observed in LLD. Above, we described the large-scale neural networks that contribute to clinical symptoms in LLD. A growing literature indicates that decreased activity in the ECN and hyperactivity in the SN and DMN are associated with some of the most prominent symptoms in LLD (for example, executive dysfunction, anxiety, and apathy). Furthermore, recent evidence suggests that resting-state network activity can distinguish several neural endophenotypes of major depression with distinct clinical symptom profiles. Drysdale *et al*. ^76^ reported that varying patterns of abnormal FC differentiated four neurophysiological subtypes in middle-aged patients with major depression. Two subtypes demonstrated greatly reduced FC in DMN and SN, and two subtypes demonstrated reduced FC in ECN and DMN regions. Patients with reduced DMN-SN FC exhibited more vegetative symptoms, whereas patients who exhibited reduced ECN-DMN connectivity had greater anxiety. Although similar analyses have yet to be conducted with LLD, Li *et al*. found that different symptoms correlated with distinct inter-network connectivity patterns determined within independent components analysis ^67^.

The traditional clinical diagnostic entity of major depression very likely represents several diseases and distinct syndromes. It is plausible that at least some of the contradictory fMRI findings in the literature on LLD or major depressive disorder (for example, studies reporting divergent FC of the same networks) occur as a result of patient cohorts being recruited and lumped together as one disorder based upon traditional descriptive diagnostic systems. This underlies the importance of disentangling the neuroanatomical substrates of clinical symptoms in LLD. If the studies mentioned above are used as a foundation, neurobiologically informed subtypes of LLD may soon serve as precise guides to identifying disease mechanisms. Furthermore, together with other imaging modalities (for example, amyloid PET), fMRI may be especially useful in clarifying the overlap between LLD and neurodegenerative disorders.
